# Exceptional long-term responses from OCEAN and HORIZON trials: melflufen-dexamethasone as an expansion of treatment options for relapsed/refractory multiple myeloma in the era of new immunotherapies?

**DOI:** 10.1007/s00432-025-06326-3

**Published:** 2025-10-09

**Authors:** M. Talarico, S. Barbato, V. Maisnar, S. Delimpasi, M. Puppi, I. Rizzello, L. Pantani, P. Tacchetti, M. Martello, I. Vigliotta, C. Terragna, M. Cavo, Elena Zamagni, K. Mancuso

**Affiliations:** 1 IRCCS Azienda Ospedaliero-Universitaria di Bologna, Istituto di Ematologia “Seràgnoli”, Bologna, Italy; 2 Dipartimento di Scienze Mediche e Chirurgiche, Università di Bologna, Via Massarenti, 9, 40138 Bologna, Italy; 3 Charles University Hospital and Faculty of Medicine, Hradec Kralove, Czech Republic; 4 Hematology Clinic and BMT Unit, Evangelismos Hospital, Athens, Greece

**Keywords:** Multiple myeloma, Melflufen, Peptide-drug conjugate, Long-term responders

## Abstract

Alkylating agents have represented the first effective drug class in multiple myeloma (MM) but, since the introduction of novel effective drugs, their use has progressively decreased and is currently relegated to autologous stem cell transplant (ASCT) and few other settings. Nevertheless, the combination of melflufen (a peptide-drug conjugate pro-drug of melphalan) and dexamethasone was approved by the U.S. Food & Drug Administration (FDA) for triple-class refractory (TCR) patients after ≥ 4 prior lines of therapy (LOT) following results of HORIZON clinical trial (NCT02963493). This combination was subsequently withdrawn as it was not associated with improved overall survival (OS) as compared to pomalidomide-dexamethasone (OCEAN clinical trial, NCT03151811). However, since a post-hoc analysis showed a benefit in OS for patients without prior ASCT or with a time to progression (TTP) > 36 months after ASCT, the European Medicines Agency (EMA) has approved melflufen-dexamethasone for TCR patients after ≥ 3 LOT, including specification that TTP must be ≥ 3 years in patients with prior ASCT. In this paper, we report three cases of patients receiving the combination melflufen-dexamethasone in the aforementioned clinical trials in three hematologic centers across Europe and achieving exceptionally long responses as compared to the overall enrolled populations, with good tolerability. Further, we discuss the potential use of this chemotherapy-based regimen in the era of novel immunotherapies.

## Introduction

Multiple myeloma (MM) is a hematologic malignancy characterized by clonal proliferation of malignant plasma cells (PC) within the bone marrow or in extramedullary sites. Alkylating agents have represented the first effective drug class in treatment of MM, due to intrinsic sensitivity of tumor PCs: the combination of melphalan and prednisolone (MP) has been a standard regimen for decades since its introduction in the 1960s (Alexanian et al. 1969) and constituted the backbone for novel combinations, whereas the advent of high-dose melphalan (HDM) followed by autologous stem cell transplant (ASCT) in the 1980s has revolutionized therapy of MM (Barlogie et al. 1987) and still represents a standard-of-care (SOC) procedure for transplant-eligible patients (Dimopoulos et al. 2025).

During the last two decades, the introduction of novel drugs and combinations has represented a further innovation, leading to a significant advance in patients’ outcomes. Particularly, proteasome inhibitors (PIs), immunomodulatory drugs (IMiDs) and CD38-directed monoclonal antibodies (moAbs) have drastically improved the depth of hematologic responses and outcomes, with a median overall survival (OS) exceeding 8.5 years in the real-world setting and reaching almost 12 years in patients receiving combinations of at least two novel agents (Puertas et al. 2023). Since the advent of new triplet and quadruplet regimens, the use of melphalan in MM has been progressively relegated to the ASCT setting (Dimopoulos et al. 2025). Conversely, cyclophosphamide has represented a backbone of induction regimens, is part of lymphodepleting chemotherapy before chimeric antigen receptors T-cell therapy (CAR T-cell therapy), of peripheral blood stem cells mobilization procedure, and of intensive regimens for rapid debulking of aggressive diseases (Costa et al. 2023).

Recent years have been characterized by the introduction of modern immunotherapeutic approaches, including antibody–drug conjugates (ADC) and T-cell engaging immunotherapies (CAR T-cell therapies and bispecific antibodies) (Tacchetti et al. 2024). Beside these novel treatments, a pro-drug of the classic alkylating agent called melphalan flufenamide (melflufen) has been introduced.

Melflufen is a first-in-class peptide-drug conjugate (PDC) which is rapidly internalized by malignant PCs because of its high lipophilicity. Inside the tumor cells, it undergoes the action of aminopeptidases, with subsequent selective release of the alkylating agent, which remains trapped within the cell due to its hydrophilicity, resulting in high intracellular concentration. In the absence of clinical head-to-head comparisons, in vitro studies have demonstrated a significant potency increase and better efficacy as compared to melphalan, and no added toxicities have been observed. Wickström et al. (2017). In 2021, the combination of this drug and dexamethasone was given accelerated approval by the U.S. Food and Drug Administration (FDA) in relapsed/refractory MM (RRMM) patients who had been previously treated with at least 4 lines of therapy (LOT) and were triple-class refractory (TCR), following results of the pivotal phase II HORIZON trial (NCT02963493).

This trial enrolled 157 patients having previously received at least 2 LOT (including a PI and an IMiD), refractory to pomalidomide and/or a CD38-directed moAb. In a heavily pretreated and poor-risk population (5 median previous LOT, 76% TCR, 38% with high-risk cytogenetics and 35% with extramedullary disease, EMD), this combination led to an overall response rate (ORR) of 29% (26% in TCR patients) with median duration of response (DOR) of 5.5 months (4.4 months in TCR patients). In the all-treated population median progression-free survival (PFS) and OS were 4.2 and 11.6 months, respectively; in TCR patients, median PFS and OS were 3.9 and 11.2 months, respectively. Among responders, median PFS was 8.5 months (both in the all-treated and TCR populations) and median OS was 17.6 and 16.5 months in the all-treated and TCR populations, respectively. Hematologic toxicity represented the most common grade ≥ 3 adverse event (neutropenia in 79%, thrombocytopenia in 76% and anemia in 43% of patients), whereas pneumonia was the most common grade ≥ 3 non hematologic event (10% of patients); gastrointestinal adverse events occurred in 62% of patients and were mostly grade < 3 (93%) (Richardson et al. 2021). Efficacy of this combination was also confirmed by subgroup analyses of patients with high-risk cytogenetics or EMD (Mateos et al. 2020; Richardson et al. 2020).

Subsequently, the phase III OCEAN trial (NCT03151811) randomized 495 lenalidomide-refractory patients (2–4 prior LOT including lenalidomide and a PI) to receive melflufen-dexamethasone or pomalidomide-dexamethasone (Pd), showing a benefit of ORR (33% vs. 27%, *p* = 0.16) and median PFS (6.8 vs. 4.9 months, HR = 0.79, *p* = 0.032) but worse median OS (20.2 vs. 24 months, HR = 1.1, *p* = 0.24) (Schjesvold et al. 2022). Due to the OS data in the intention-to-treat population, melflufen was withdrawn in the USA. However, further analyses revealed a significant difference in OS between patients without previous ASCT or with a time to progression (TTP) > 36 months after ASCT and patients with a TTP < 36 months after ASCT: in the former group, median OS was 23.6 months with melflufen-dexamethasone and 19.8 months with Pd (HR = 0.83, *p* = 0.225); in the latter group, median OS was 15.7 months with melflufen-dexamethasone and 28.7 months with Pd (HR 1.8, *p* = 0.033). When excluding patients who had progressed < 36 months after HDM, ORR was 42% vs. 26%, median PFS was 9.3 vs. 4.6 months (HR = 0.58, *p* = 0.0001) and median OS was 23.6 vs. 19.8 months (HR = 0.83, *p* = 0.22). A similar post hoc analysis from HORIZON showed a significant benefit of ORR (29% vs. 22%), median DOR (7.6 vs. 3.9 months) and median PFS (4.2 vs. 3.4 months) for patients without previous ASCT or with TTP > 36 months after ASCT (Sonneveld et al. 2023).

Despite these data, FDA confirmed withdrawal of approval of melflufen from U.S. market (FDA 2024), whereas the European Medicines Agency (EMA) has approved the combination melflufen-dexamethasone for the treatment of TCR patients who have received at least 3 previous LOT, with disease progression on or after last therapy, including specification that TTP must be ≥ 3 years in patients with prior ASCT (EMA 2022). Table 1 summarizes efficacy and safety of HORIZON and OCEAN.

**Table 1:** Main characteristics of "HORIZON" and "OCEAN" clinical trials

Characteristics	HORIZON		OCEAN			
	Overall population (N = 157)	Indicated population (N = 52)	Overall population		Indicated population	
			Md (N = 246)	Pd (N = 249)	Md (N = 145)	Pd (N = 148)
Median age, ys	65	70	68	68	UR	
Median no of prior LOT	5	5	3	3	UR	
Prior ASCT, no (%)	108 (69)	19 (37)	125 (51)	120 (48)	UR	
TCR pts, no (%)	119 (76)	52 (100)	39 (16)	30 (12)	UR	
EMD, no (%)	55 (35)	22 (42)	31 (13)	31 (12)	UR	
HR cytogenetics, no (%)	59 (38)	21 (40)	83 (34)	86 (35)	UR	
ORR, %	29.3	28.8	32.5	26.9	42.1	26.3
≥ VGPR, %	10.8	9.6	12.2	8.4	18.6	8.1
Median PFS, mos	4.2	4.2	6.8	4.9	9.3	4.6
Median OS, mos	11.6	11.2	20.2	24.0	23.6	19.8
Neutropenia, no (%) G ≥ 3, no (%)	129 (82) 124 (79)	UR	137 (60) 24 (54)	113 (46) 02 (41)	UR 91 (66)	UR 75 (51)
Anemia, no (%) G ≥ 3, no (%)	111 (71) 67 (43)	UR	152 (62) 97 (42)	92 (37) 44 (18)	UR	
Thrombocytopenia, no (%) G ≥ 3, no (%)	129 (82) 120 (76)	UR	160 (70) 143 (63)	48 (19) 6 (11)	UR 96 (70)	UR 17 (12)

Richardson et al. (2021), Schjesvold et al. (2022), Sonneveld et al. (2023), EMA (2025)

Indicated population = pts without previous ASCT or with TTP ≥ 36 mos after ASCT; high-risk cytogenetics is defined in presence of del(17p), t(4;14), t(14;16), t(14;20) or gain(1q).

Abbreviations: Md = melflufen + dexamethasone; Pd = pomalidomide + dexamethasone; pts = patients; no number; ys = years; LOT = lines of therapy; ASCT = autologous stem cell transplant; TCR = triple-class refractory; EMD = extramedullary disease; HR = high-risk; ORR = overall response rate; VGPR = very good partial response; mos = months; G = grade; UR = unreported; TTP = time to progression.

Herein, we report three cases of RRMM patients enrolled in the aforementioned clinical trials in three hematologic centers across Europe (Bologna, Athens, Hradec Kralove), achieving exceptionally long responses with the combination melflufen-dexamethasone as compared to the overall enrolled populations.

## Case 1: melflufen-dexamethasone as eighth-line of therapy in a TCR-ASCT exposed patient

A 61-year-old man was diagnosed with IgG/kappa MM at the UOC Ematologia, IRCCS Azienda Ospedaliero-Universitaria di Bologna (Italy) in February 2010. MM was active for anemia. International Staging System (ISS) was I and fluorescence in situ hybridization (FISH) analysis was negative for IMWG high-risk features. The patient resulted primary refractory to bortezomib-dexamethasone (Vd) and to vincristine-doxorubicin-dexamethasone, then received lenalidomide-dexamethasone (Rd) as third-line treatment followed by tandem ASCT, achieving a very good partial response (VGPR) (Kumar et al. 2016) with a TTP of 28 months from ASCT. The patient then resulted refractory to Pd, elotuzumab-Rd and bendamustine-Vd before reaching a partial response (PR) with single-agent daratumumab, which was administered for a total of 28 cycles. After biochemical relapse and subsequent newly-onset anemia, this TCR patient was enrolled in the HORIZON clinical trial at the age of 70 years. Bone marrow biopsy revealed 90% of PCs and skeletal assessment by 18F-FDG-PET/CT was negative for bone disease/EMD. Administration of melflufen-dexamethasone began in July 2019. Because of hematologic toxicity (grade 2 anemia requiring transfusions, grade 4 neutropenia requiring Granulocyte Colony-Stimulating Factor support and grade 4 thrombocytopenia), causing frequent scheduling delays, dose was reduced from 40 to 30 mg from the fourth cycle and to 20 mg thereafter. Other adverse events included grade 2 fatigue and grade 2 infections involving urinary tract and upper airways, whereas the patient did not experience any gastrointestinal toxicity. A PR was achieved after about one year of treatment and dose modifications allowed to maintain good hematological values. After approximately four years of therapy, trilinear cytopenia progressively developed (nadir values: hemoglobin 9 g/dL, platelet count 45000/mmc, absolute neutrophil count 880/mmc). Bone marrow examination showed dysplastic morphology of erythropoiesis and megakaryopoiesis with blast cells < 2%. Karyotype analysis showed deletion of 7q and gain of 11q chromosomes, whereas FISH analysis displayed loss of KMT2E and EZH2 loci and duplication of KMT2A locus. Myeloid next generation sequencing (NGS) panel revealed the presence of variants in DNMT3A and CSF3R, deletions involving BRAF and EZH2 and two pathogenic single-nucleotide variants with low variant allele frequency (1.5%) in TP53. Therefore, a diagnosis of myelodysplastic neoplasm (MDS) with low blasts and intermediate risk Revised International Prognostic Scoring System (IPSS-R) was made (Khoury et al. 2022; Greenberg et al. 2012). Due to the onset of this second primary malignancy (SPM), treatment was discontinued from August 2023, after a total of 46 cycles administered. During subsequent follow-up, a biochemical progression of MM also occurred from December 2023 (PFS of 53.5 months), but no subsequent anti-myeloma therapy was started due to comorbidities, including the hematological SPM.

## Case 2: melflufen-dexamethasone as third-line of therapy in an ASCT non-exposed patient

A 57-year-old man was diagnosed at Charles University Hospital and Faculty of Medicine, Hradec Kralove (Czech Republic) in January 2017 with IgG/lambda MM, active for anemia, bone disease, renal insufficiency and hypercalcemia. Baseline cytogenetic analysis showed the presence of hyperdiploid karyotype and absence of HR chromosomal abnormalities; ISS was III and Revised-ISS (R-ISS) was II. The patient was treated with a triplet combination based on bortezomib, thalidomide and dexamethasone (VTd) for 8 cycles achieving a VGPR (the patient refused to undergo ASCT). After a biochemical progression, the patient received Rd, achieving a PR. After a further biochemical progression, the patient was enrolled in the OCEAN trial and randomized to receive melflufen-dexamethasone as third-line treatment. Therapy administration began in September 2019 at 40 mg starting dose. Because of hematologic toxicity (grade 3 anemia, grade 4 thrombocytopenia and grade 2 neutropenia), dose was reduced to 30 mg from the twelfth cycle and 20 mg from the thirteenth cycle. Other adverse events included a grade 3 pneumonia and grade 1 fatigue. After 4 cycles of therapy, a complete response (CR) was achieved. After 57 cycles, treatment is still ongoing with a hematologic CR.

## Case 3: melflufen-dexamethasone as third-line of therapy in an ASCT exposed patient

A 41-year-old man was diagnosed with IgG/lambda MM in July 2012 at the Hematology Clinic and BMT Unit, Evangelismos Hospital, Athens (Greece). MM was active for anemia and acute kidney injury. FISH analysis was negative for high-risk features; ISS and R-ISS were II. The patient received first-line treatment with bortezomib-cyclophosphamide-dexamethasone (VCd) with subsequent relapse. The second-line treatment was represented by Rd, which was administered for 2 cycles with achievement of a PR and followed by ASCT (achieving a VGPR) and by Rd maintenance. TTP from ASCT was 59 months. After a biochemical relapse, this double-refractory patient was enrolled in the OCEAN clinical trial and randomized to receive melflufen-dexamethasone. Bone marrow biopsy revealed 20% of plasma cells and skeletal assessment by whole-body CT was negative for bone disease. Therapy was started in January 2018 at 40 mg. Due to hematologic toxicity (grade 1 anemia and thrombocytopenia, grade 2 neutropenia) dose was reduced to 30 mg from the twelfth cycle. The patient also experienced grade 2 fatigue, grade 1 nausea and grade 1 upper airways infection. After 4 years of therapy, a stringent CR was achieved. Treatment is still ongoing after 83 cycles.

## Discussion

Despite the introduction of several novel drugs, MM is still considered an incurable disease, with the majority of patients relapsing and requiring further LOT even though achieving deep and prolonged responses and even minimal residual disease negativity. Treatment of RRMM represents a considerable challenge, with achievement of dismal survival as the disease becomes refractory to standard drug classes. In triple-class exposed (TCE) and TCR patients, a median PFS of 4.6 and 3.9 months and a median OS of 13.8 and 11.1 months have been respectively documented (Mateos et al. 2024), encouraging the need for new treatment strategies with diverse mechanisms of action.

Recent years have been characterized by the introduction of novel drugs, particularly BCMA- and GPRC5D-directed T-cell engaging immunotherapies (CAR T-cell and bispecific antibodies). Both idecabtagene autoleucel (ide-cel) and ciltacabtagene autoleucel (cilta-cel) were first approved for TCE patients having received at least 3 (EMA) or 4 (FDA) prior LOT. These approvals are based on KarMMa and CARTITUDE-1, which showed ORR of 73–97% (≥ CR and MRD negativity in 33–67% and 26–34% of patients, respectively), median PFS and OS of 8.8 and 24.8 months for ide-cel, and of 34.9 months and 60.7 months for cilta-cel (with one third of patients remaining alive and progression-free for ≥ 5 years) in heavily pretreated populations (median of 6 prior LOT, more than 80% TCR patients) (Munshi et al. 2021; Lin et al. 2023; Berdeja et al. 2021; Martin et al. 2023; Jagannath et al. 2025). Further, ide-cel was approved in TCE patients previously treated with at least 2 prior LOT and cilta-cel in lenalidomide-refractory patients after at least 1 prior LOT as they were shown to improve response rates and outcome as compared to SOC regimens (Rodriguez-Otero et al. 2023; Ailawadhi et al. 2024; San-Miguel et al. 2023). Moreover, four bispecific antibodies (teclistamab, elranatamab, linvoseltamab and talquetamab) were recently approved for TCE patients having received at least 3 (EMA) or 4 (FDA) prior LOT, as they showed ORR ranging from 61 to 74%, with 33% to 49% of patients achieving ≥ CR and median PFS of 7.5 to 17.2 months in heavily pretreated populations (medians of 5–6 prior LOT, with TCR patients ranging from 75 to 97% across different trials) (Moreau et al. 2022; Garfall et al. 2024; Lesokhin et al. 2023; Miles Prince et al. 2024; Bumma et al. 2024; Chari et al. 2022; Chari et al. 2025).

Due to the aforementioned results, novel immunotherapies have revolutionized the therapeutic scenario of RRMM and, from the efficacy standpoint, are among the best therapeutic options that can be offered to TCR patients. In absence of randomized trials, a recent matched-adjusted indirect comparison has shown that patients receiving cilta-cel have a 3.8-fold improvement of ORR and are 186-fold more likely to achieve ≥ CR with a statistically significant improvement of PFS and OS as compared to patients receiving melflufen-dexamethasone (Weisel et al. 2022). However, long DOR may be achieved in patients responding to the PDC, especially in patients without prior ASCT or having a TTP > 36 months from HDM (Sonneveld et al. 2023), as our cases highlighted (despite patient 1 had a TTP of 28 months from ASCT). Beyond efficacy, the use of T-cell engaging therapies harbors issues regarding accessibility, high costs (Keesari et al. 2025) and safety. Indeed, a careful selection of patients is necessary, as several toxicities have been described in pivotal clinical trials and confirmed in real-life experiences. Particularly, cytokine release syndrome (CRS) and neurotoxicity have been reported in up to 95% (grade ≥ 3 in up to 5%) and in up to 18% of patients (grade ≥ 3 in up to 3%) respectively, usually requiring hospitalization for CAR-T infusion and step-up doses of bispecific antibodies. Moreover, grade ≥ 3 neutropenia and thrombocytopenia persisting > 1 month have been reported in up to 41% and 48% of patients receiving BCMA-directed CAR T-cell therapies across clinical trials and grade ≥ 3 infections in up to 55% of patients receiving novel immunotherapies, particularly bispecific antibodies (Munshi et al. 2021; Berdeja et al. 2021; Martin et al. 2023; Rodriguez-Otero et al. 2023; Ailawadhi et al. 2024; San-Miguel et al. 2023; Moreau et al. 2022; Garfall et al. 2024; Lesokhin et al. 2023; Miles Prince et al. 2024; Bumma et al. 2024; Chari et al. 2022; Chari et al. 2025; Mancuso et al. 2025a, 2025b), thus making necessary antimicrobial prophylaxes, patient compliance regarding vaccination strategies and immunoglobulin replacement therapy, which have been associated to a tenfold decrease of serious infections in this setting (Lin et al. 2024; Ludwig et al. 2023; Rodriguez-Otero et al. 2024; Lancman et al. 2023; Frerichs et al. 2024; Mancuso et al. 2025a, 2025b).

Aforementioned toxicities may limit use of T-cell engaging immunotherapies in clinical practice, especially for elderly, comorbid or frail patients, though some recent experiences have shown a potential feasibility in this setting (Davis et al. 2024; Adegbite et al. 2025). On the other hand, melflufen-dexamethasone represents a well-tolerated combination, with infectious events being relatively rare and hematologic toxicities representing the most common grade ≥ 3 adverse event both in clinical trials and in the real-world setting (Richardson et al. 2021; Schjesvold et al. 2022; Hossain et al. 2025), though they can be managed with adequate supports, dose delays or reduction, as in above reported cases.

Furthermore, even though the PDC was administered via central venous catheter in the pivotal trials, the PORT study (NCT04412707) showed that administration via peripheral venous catheter is bioequivalent and well tolerated, thus potentially reducing the risk of infections and thrombosis. Pour et al. (2024) Moreover, melflufen may be administered in patients with moderate renal impairment, as the phase 2 BRIDGE trial (NCT03639610) did not show new safety signals in patients with an estimated glomerular filtration rate (eGFR) between 30 and 45 mL/min/1.73 m^2^, even though exposure to melphalan increases in patients with reduced eGFR (Pour et al. 2021), therefore a dose of 30 mg in place of 40 mg is recommended in this population (EMA 2025). Subgroup analyses of elderly patients receiving this treatment in the HORIZON trial revealed that both efficacy and toxicities in patients aged ≥ 75 years are consistent with data of the overall population (Larocca et al. 2020). In addition, it was shown that treatment with melflufen-dexamethasone can preserve health-related quality of life with significant improvement of global health status, physical and emotional functioning, fatigue and pain, with a substantial delay of patient-reported outcome deterioration in responding patients as compared to non-responders (Larocca et al. 2022). A serious adverse event occurring in one of the cases reported is the onset of a SPM, particularly a MDS. Development of SPMs was rarely reported across clinical trials using melflufen (Richardson et al. 2021; Schjesvold et al. 2022). However, it should be noted that our patient had received tandem ASCT and three IMiD-based LOT during his previous treatment history and a mutagenic effect of alkylating agents (especially of melphalan) and lenalidomide, with an increased risk of SPMs (particularly therapy-related myeloid neoplasms, t-MN) is well-recognized (Musto et al. 2017; Rosenberg et al. 2021; McCarthy et al. 2017). Furthermore, the onset of SPMs has been recently described following novel immunotherapies, particularly CAR T-cells (Lin et al. 2023; Martin et al. 2023; Vainstein et al. 2023). The role of baseline conditions, e.g., a clonal hematopoiesis of indeterminate potential (CHIP), as risk factors for t-MN is currently being investigated (Soerensen et al. 2020; Awada et al. 2024; Gustine et al. 2025; Avigan et al. 2025) and may help balancing expected risks and benefits and choosing the most appropriate treatment for each patient.

On top of the head-to-head choice, even in the lack of strong published data, it could be envisioned that melflufen-dexamethasone may be used also in patients eligible for modern immunotherapies, in those developing resistance to BCMA or GPRC5D-directed T-cell engaging therapies, between one and another treatment or as bridging therapy. In this regard, it should be acknowledged that the use of melflufen should be avoided in proximity of lymphocyte apheresis pre-CAR-T, as alkylating agents can affect peripheral lymphocyte count and T-cell fitness for CAR T-cell manufacturing (Lin et al. 2023; Rytlewski et al. 2022; van de Donk et al. 2025).

Beside the aforementioned immunotherapies, the ADC belantamab mafodotin (belamaf) had been previously approved for TCR patients having received at least four prior LOT following results from the pivotal DREAMM-2 trial: in the cohort receiving the approved dose of 2.5 mg/kg, with a median number of 6 prior LOT, ORR was 32% (19% ≥ VGPR), median PFS was 2.8 months (14 months in patients achieving ≥ VGPR) with low rates of grade ≥ 3 adverse events, except for ocular toxicity (Nooka et al. 2023). Similar efficacy results and good tolerability would make belamaf a competitor to melflufen-dexamethasone for TCR patients resulting ineligible to T-cell engaging therapies. However, FDA has recently withdrawn the license for the ADC (FDA 2025) and EMA did not renew its conditional marketing approval in this setting (EMA 2024) as the confirmatory phase 3 trial comparing this drug to Pd did not meet its primary endpoint of improving PFS (Dimopoulos et al. 2023). Conversely, a potential competitor to melflufen-dexamethasone is represented by selinexor-dexamethasone (Seld), as the STORM clinical trial showed ORR of 26%, median PFS of 3.7 months and median OS of 8.6 months in a heavily pretreated population (7 median prior LOT, 100% TCR, 68% penta-refractory) with good tolerability (Chari et al. 2019). However, this treatment strategy is currently approved either in an even more advanced setting (penta-refractory patients after ≥ 4 prior LOT), or from second line in combination with bortezomib (SelVd) following the BOSTON clinical trial (Grosicki et al. 2020).

The recently updated evidence-based guidelines for MM treatment published by the European Hematology Association and the European Myeloma Network suggest melflufen (in cases without prior ASCT or with TTP ≥ 3 years from ASCT) or Seld as therapeutic options for TCR patients, particularly if no other strategy is available (Dimopoulos et al. 2025). The current European approval and recommendation for the use of melflufen-dexamethasone are based on the significant benefit in patients without prior ASCT or with long (≥ 3 years) TTP from HDM (target population), as shown in post-hoc analyses of the HORIZON and OCEAN studies. Furthermore, a later subgroup analysis from OCEAN regarding the overall alkylator-refractory group (including cyclophosphamide, standard-dose melphalan, HDM and bendamustine) showed a significant benefit of melflufen versus the comparator arm only in the target population but irrespective of the refractoriness to standard dose alkylating agents. These observations have led to the hypothesis that the negative prognostic effect of TTP < 3 years after ASCT is due to the disease’ intrinsic resistance (with persistence of some tumor clones being insensitive even to high intracellular concentrations of melphalan) and to the fact that the myeloablative conditioning regimen may negatively affect the bone marrow microenvironment and the hematopoietic stem cell reserve. Conversely, having received a previous alkylator therapy outside of the ASCT setting may not impact the efficacy of the PDC, thus suggesting the non-completely super-imposable mechanism of action of melflufen as compared to other alkylating agents, also supported by in vitro studies (Wickström et al. 2017). Nonetheless, it should be highlighted that clinical head-to-head comparisons between the PDC and other alkylating agents are lacking (Sonneveld et al. 2023; Schjesvold et al. 2024; Kapoor and Gonsalve 2022). Furthermore, clinical data regarding the use of other alkylators in a similar setting as the current European approval of melflufen are also lacking or outdated (Costa et al. 2023), though second-line ASCT can be considered a potential therapeutic option in patients with TTP ≥ 3 years from prior HDM and lenalidomide-maintenance with limited access to novel more efficacious regimens (Dimopoulos et al. 2025).

Melflufen-dexamethasone has also been investigated in combination with daratumumab or bortezomib in the phase 1/2a ANCHOR trial (NCT03481556), showing encouraging efficacy results and manageable toxicities (Ocio et al. 2024). Subsequently, the phase 3 trial LIGHTHOUSE compared the former combination to the doublet without melflufen in patients resulting refractory to a PI and an IMiD or having received at least three prior LOT including a PI and an IMiD. Even though the trial had a premature closure due to a partial clinical hold by FDA for all melflufen studies, it showed a benefit of ORR (59% vs. 30%) and median PFS (not reached with a median follow-up of 7.1 months vs 4.9 months), especially in patients without prior ASCT or with TTP > 36 months from ASCT, with hematologic toxicities representing the most common adverse events (Pour et al. 2024).

Limitations of our case reports are the absence of HR features (no patient presented EMD or cytogenetically defined high-risk disease, even though patient 1 had a functional high-risk due to primary refractoriness to former LOT), heterogeneity regarding prior LOT and absence of quadruplet regimens (representing the actual SOC) in treatment history. Furthermore, it should be acknowledged that our cases were retrospectively selected among the ones with longest responses in the clinical trials, with extraordinary results as compared to the overall enrolled populations and to average outcomes in the real-world setting. For example, among 15 patients receiving melflufen-dexamethasone in Bologna (4 median prior LOT, 87% TCR, 40% penta-refractory), ORR was 47% (CR in 14%) and median PFS was 4 months.

In conclusion, the three clinical cases presented here showed unexpected and out of average long-term responses with the combination of melflufen and dexamethasone. Certainly, the exceptional nature of these cases compared to the overall population of patients involved in the same clinical trials must be underlined, and the limitations discussed here must be considered. Furthermore, the advent of modern immunotherapies in the same setting has led to unprecedent superior and durable responses. Nonetheless, the availability of the latter therapies may differ across countries due to local health policies, logistical issues, economic reasons and, not least, their safety profile and management issue, making CAR T-cell therapies and bispecific antibodies unsuitable for all patients. Conversely, melflufen may balance a fair amount of efficacy (with similar results as compared to Seld or belamaf), safety and quality of life, especially in cases without prior ASCT or with long TTP from ASCT, as the reported cases confirmed. Moreover, the use of melflufen in less heavily pretreated populations or in combination strategies with other drugs should be further investigated. Overall, melflufen may remain a valid option for TCR elderly patients, for those resulting unsuitable for T-cell engaging immunotherapies, imbricated within or after failure of immunotherapies, or in countries where these novel therapies are not available yet.

## Data Availability

All data generated or analyzed during this study are included in this article. Further inquiries can be directed to the corresponding author.
